# Trustworthiness Measurement Algorithm for TWfMS Based on Software Behaviour Entropy

**DOI:** 10.3390/e20030195

**Published:** 2018-03-14

**Authors:** Qiang Han

**Affiliations:** 1 School of Computer Science and Engineering, North Minzu University, Yinchuan 750021, China; hanqiang@bupt.edu.cn; Tel.: +86-139-9501-3650; 2 Key Laboratory of Trustworthy Distributed Computing and Services (Ministry of Education), Beijing University of Posts and Telecommunications, Beijing 100871, China

**Keywords:** software behaviour trustworthiness, principle of maximum entropy, measurement algorithm, workflow management system

## Abstract

As the virtual mirror of complex real-time business processes of organisations’ underlying information systems, the workflow management system (WfMS) has emerged in recent decades as a new self-autonomous paradigm in the open, dynamic, distributed computing environment. In order to construct a trustworthy workflow management system (TWfMS), the design of a software behaviour trustworthiness measurement algorithm is an urgent task for researchers. Accompanying the trustworthiness mechanism, the measurement algorithm, with uncertain software behaviour trustworthiness information of the WfMS, should be resolved as an infrastructure. Based on the framework presented in our research prior to this paper, we firstly introduce a formal model for the WfMS trustworthiness measurement, with the main property reasoning based on calculus operators. Secondly, this paper proposes a novel measurement algorithm from the software behaviour entropy of calculus operators through the principle of maximum entropy (POME) and the data mining method. Thirdly, the trustworthiness measurement algorithm for incomplete software behaviour tests and runtime information is discussed and compared by means of a detailed explanation. Finally, we provide conclusions and discuss certain future research areas of the TWfMS.

## 1. Introduction

The workflow management system (WfMS) is considered a multidisciplinary system-of-systems (SoS)-oriented software under the modern IT background of cloud computing, internet of things (IoT), big data, and advanced technologies for the future. When a WfMS encounters unexpected accidents, human intervention or offline adjustment is insufficient to be appropriated for its complex undertaken commissions and high availability requirements. Similar to real-time systems, a WfMS requires online adjustment to accommodate random changes occurring in its surroundings or software architecture under human control strategies, which may affect its functionalities or non-functionalities and transaction data consistence—and even its trustworthiness level. For example, addressing course timetabling problems [1] from the case-based reasoning (CBR) system viewpoint, to adapt to the limited classroom capacity for the ever-increasing number of students, the CBR system may change the venue to a new classroom with larger capacity to prevent basic teaching functionality from being interrupted. In addition, to adapt to enable more effective teaching, the CBR system may adjust the venue to a new classroom that better suits the preference of the teacher and students to optimise the potential teaching non-functionality. Furthermore, to adapt to achieve more consistent teaching transactions log data, the CBR system may recover or repair the transactions with a uniform state; otherwise, the complete student achievement would not be generated from the conflicting data. Although the CBR system works for education-oriented business process management tasks, i.e., a special type of WfMS, the random changes mentioned above could mislead the WfMS software behaviour and eventually directly or indirectly affect the WfMS service behaviour realised by its software, ultimately reducing the WfMS software trustworthiness and resulting in users abandoning the candidate WfMS adoption. In order to solve the abovementioned problem, we present a novel measurement algorithm based on previous research results. Our approach includes a self-automatic framework for random changes and relative algorithms, concentrating on measuring the similarity between software behaviour and its claims through black-box testing with incomplete software behaviour entropy. The remainder of this paper consists of three sections: Section 2 discusses the related work on measurement methods regarding the WfMS; Section 3 illustrates the novel measurement algorithm from two perspectives, namely the calculus implemented on the WfMS and the normal forms of the WfMS; and Section 4 discusses the proposed algorithm and summarises future research areas.

## 2. Related Work

Software trustworthiness [2] is considered a non-functionality combination of software quality against the conformance degree oriented to subjective user evaluation. Against this background, the difference between user requirements and actual software behaviour will determine the trustworthiness perceived by the user. In recent decades, considerable efforts have been made to design intelligence algorithms to measure the difference between objective software or service behaviour and subjective user evaluation. In references [3,4], the authors define the similarity and design based on the genes of the firing sequences generated from transition adjacency relations (TARs). The corresponding distance measures between processes were taken as a metric to be applied in artificial processes and evaluations for clustering real-life processes. As a promising candidate for further research areas, the authors of reference [5] took into account the active feedback of evaluation data in workflow process modelling, which encompassed the entire lifecycle of workflow and enabled active real-time controlling using workflow audit trail data from three perspectives, namely process, resource, and object. In order to assist users in selecting a sufficient workflow with appropriate quality of service (QoS) to meet their requirements, reference [6] proposed a novel approach to scientific workflow retrieval with cost constraints and defined a distance measure for comparing the similarities among cost constrained graph (CCGs), through which workflow retrieval and ranking could be conducted based on similarity computations. To allow companies to respond to changing markets by creating product variants derived from different combinations of existing or new modular components, reference [7] sought to define and measure workflow modularity. The authors made three contributions: they designed two important performance measures regarding flow time and flexibility; they proposed an integer nonlinear programming (INLP) optimisation model for designing modular workflows that can be adopted for small processes; and they presented a heuristic model for the same commission adopted for larger processes. From a workflow standardisation perspective, in order to translate business data into bytes that are consumable by daily information systems with less automation difficulty and more reliability, as well as to make business competition comparisons more precise and invariable, reference [8] focused on health care data and workflow, reengineering the coding workflow and performance benchmarks, and proposed the establishment of useful tools for improving data quality, which can be used by everyone. In terms of the practical computation environment known as grid computing, reference [9] layered the clustering approach of the reusable workflow into a hierarchical model consisting of activity, event, condition-action, rule, and process similarity measures based on event condition action event condition action (ECA) theories. In the industry-integrated manufacturing environment, digital printing should eliminate redundant processes in order to shorten press runs and save costs. In order to select equipment and software for supporting such workflows, reference [10] introduced a workflow configuration tool that can elicit customer printing requirements and classified the requirements into a set of equivalent workflows according to their configuration using a compression-based dissimilarity measure (CDM) approach. The continuous improvement of business process management (BPM) is an ongoing issue for the WfMS, and in order to address this, a solid understanding of the success elements and waste experience during the same workflow process is essential. With the trend of lean production, reference [11] surveyed research and presented a conceptualisation for understanding the workflow and simultaneously occurring waste in the production of buildings through the three different dimensions of smoothness (high level of direct work), quality, and intensity. Moreover, they summarised the methods for this purpose, consisting of an integrated method of observation and self-reporting, as well as the last planner system (LPS) based on the method to measure workflow as handover of work between trades.

From an overview of the above related works, we can draw the conclusion that BPM measurement can be transformed from socialised human production to mechanised computing by means of electronic equipment. Therefore, there is a need for a comprehensive approach to connect the macroscopic and microscopic measurements systematically, thereby reflecting the user’s subjective experience of the trustworthiness of BPM according to the objective evidence of trustworthiness of the WfMS. However, in the dynamic and open WfMS runtime environment, the goal of comprehensively interpreting and obtaining the WfMS software behaviour is almost impossible to achieve. Generally, we can only obtain partial software behaviour, which represents incomplete trustworthiness of the entire service QoS without sufficient preciseness when the parameters regarding other software behaviour are uncertain [12]. Accordingly, we first present a quality management system (QMS) [13] of maximum entropy (QMSOME) derived from the trustworthy WfMS (TWfMS) framework, which is a thermodynamics-related interpretive model, as the virtual mirror of complex real-time business processes of the underlying information systems of organisations. Secondly, we present a novel recursive measurement algorithm for WfMS trustworthiness by means of a hybrid method derived from large-scale software black-box testing, as illustrated in the following sections, which is also an application of the principle of maximum entropy (POME) or maximum-entropy principle (MEP) [14,15].

## 3. Measurement Algorithm for the Workflow Management System Trustworthiness

In line with the related works discussed above, as a special type of QMS for workflow quality management, WfMSs experience multiple loops consisting of re-engineering and/or reorganisations. This process improves the WfMS into a high-level order—with relatively low entropy of information systems—from low-level disorder—with high entropy of information systems in the long term— along with consistently maintaining its trustworthiness as defined by users. This cycle, which covers the entire life of the WfMS, is also known as the resilience engineering (RE) [16] model and is illustrated in this section. Thereafter, we introduce its definition, including integrated manufacturing business processes, and the translation of these into models. Prior to proposing the RE model for the WfMS, we illustrate our thoughts regarding the TWfMS according to the standard WfMC reference model with the extension of methods and mechanisms, which is the fundamental goal of RE. Considering that the implementation of RE for the WfMS is ultimately imposed on the WfMS components, we first provide the preliminary component definition for the WfMS. Secondly, as the fundamental goal of WfMS RE is to assure its trustworthiness as perceived by users, we provide the trustworthy component definition of the WfMS. Thirdly, in order to certify WfMS recovery as its common function under RE, we present a normal form (NF) set [17] as a so-called paradigm to label the WfMS trustworthiness level at runtime.

### 3.1. Formal Representation of the Workflow Management System Trustworthiness

**Figure 1:**
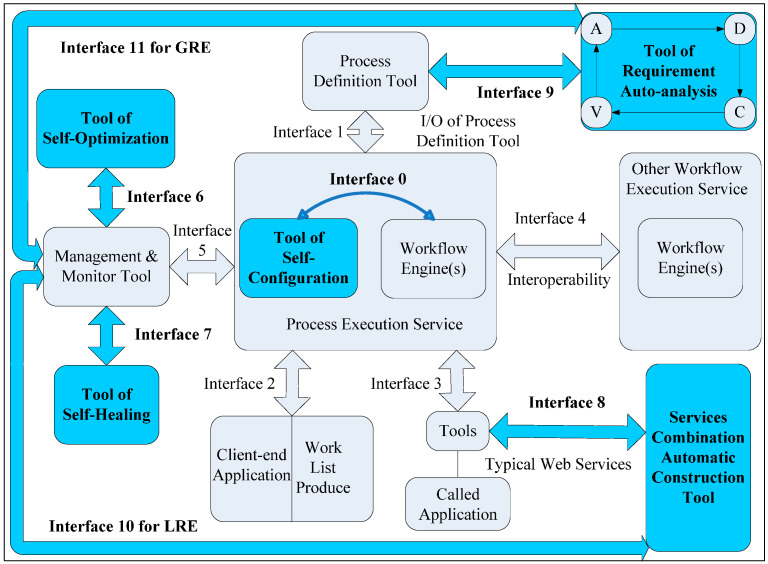
Reference model for a trustworthy workflow management system (TWfMS) with resilience engineering (RE)*.*

As SoS-oriented, complex system software, a formal representation of the mechanisms and methods revealing the internal principles of WfMS trustworthiness is imperative for the design, development, and maintenance tasks covering the entire WfMS lifecycle. In our prior research, we presented a reference model for the TWfMS [18,19], as illustrated in Figure 1, inspired by the RE concept. As indicated in Figure 1, we expand the WfMC reference model from interfaces no.0 and no.6–no.11. In the following paragraphs, we explain the differences between the TWfMS and WfMS in terms of each of these interfaces.

(1) Interface no.0 is linked to the core work engine(s) component of the WfMS and the additional self-configuration-parameter system for the WfMS (SCP4WMS) RE tool in the process execution service module; that is, we consider the SCP4WMS tools an extension of and supplementary to the process execution service module.
(2) Interfaces no.6 and no.7 are linked to the self-optimization framework system for the WfMS (SOF4WMS) and self-healing model system for WfMS (SHM4WMS) RE tools, respectively, with the management and monitoring tool, which constructs the TWfMS mechanism with the SCP4WMS tool; that is, we consider the SOF4WMS and SHM4WMS tools extensions of and supplementary to the management and monitoring tool.
(3) Interface no.8 is linked to the tools for communication on called application of typical web services with an additional tool, the auto construction method for the WfMS (ACM4WMS), based on services combination; that is, we consider the ACM4WMS tool an extension of and supplementary to the standard tools linked to the process execution service module via interface no.3.
(4) Interface no.9 is linked to the requirement auto-analysis tool with the process definition tool, where the former consists of four components known as acquisition, decomposition, combination, and verification based on a Petri net (ADCV-PN); that is, we consider the ADCV-PN tools extensions of and supplementary to the process definition tool.
(5) Interface no.10 is connected to the management and monitoring tool with the ACM4WMS tool when the WfMS encounters “local break points”, whereby the WfMS trustworthiness can no longer be maintained by the management and monitoring tool, even with the assistance of the tool sets of SCP4WMS, SOF4WMS, and SHM4WMS. In the context of the scenario described above, via interface no.10, the management and monitoring tool transfers the exceptional event unsolved by the SCP4WMS, SOF4WMS, and SHM4WMS tool sets sequentially to the ACM4WMS tool, in order to reconstruct the WfMS by searching for resources in the cloud. At such a time, we consider the WfMS as beginning local resilience engineering (LRE).
(6) Interface no.11 is connected to the management and monitoring tool with the ADCV-PN tools when the WfMS encounters “global break points”, whereby the WfMS trustworthiness can no longer be sufficiently accurate by means of the ACM4WMS tool, even if all of the resources in the cloud are traversed by means of the ACM4WMS tool. In the context of the scenario described above, via interface no.11, the management and monitoring tool finally transfers the exceptional event unsolved by the ACM4WMS tool to the ADCV-PN tools, in order to remodel the WfMS under user validation. At such a time, we consider the WfMS as beginning global resilience engineering (GRE).

**Figure 2:**
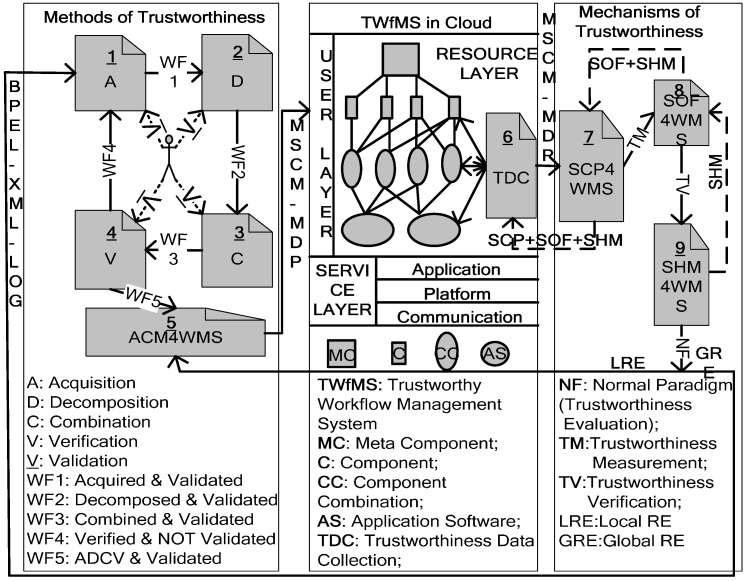
Methods and mechanisms of a TWfMS with RE.

Based on the above model, from the implementation view of software architecture, we propose the core components of the methods and mechanisms [18,19] as illustrated in Figure 2. Compared with the method of trustworthiness and the TWfMS in the cloud in Figure 2, here we place emphasis on the mechanisms of trustworthiness, comprising SCP4WMS, SOF4WMS, and SHM4WMS:

1. SCP4WMS means self-configuration-parameter system for the WfMS. It has the function of analysing the parameters transferred from the trustworthiness data collection (TDC) component, which gathers real-time data from the WfMS at the multilevel of components, component combinations, and application software. According to the analysis, SCP4WMS carries out the following procedures.
  1.1. If the parameters of the operating environment variables of workflow engines have changed and will cause WfMS failure and improper operation, SCP4WMS will modify the parameters of the WfMS itself according to predefined rules and return the new parameters of the WfMS itself to the TDC in order to be adaptable to the new environment variables of the workflow engines.
  1.2. SCP4WMS should transmit the remaining parameters to SOF4WMS to deal with other WfMS mechanisms.
  1.3. SCP4WMS should compute the WfMS behaviour trustworthiness according to the algorithm illustrated in Section 3.2 and transform it into the subsequent mechanism SOF4WMS.
  1.4. When SCP4WMS receives the new parameters of the WfMS itself, modified by SOF4WMS, it should return these to the TDC in order to be optimised with the new operating condition variables of the workflow engines.
  1.5. When SCP4WMS receives the new parameters of the WfMS itself, modified by SHM4WMS, it should return these to the TDC in order to be recovered with the new transaction consistence variables of the workflow engines.
2. SOF4WMS means self-optimisation framework system for the WfMS, and one of its functions is analysing the parameters transferred from the SCP4WMS component. According to the analysis, SOF4WMS carries out the following procedures.
  2.1. If the parameters of the operating condition variables of the workflow engines have changed and will lead to worse or better WfMS performance, SOF4WMS will modify the parameters of the WfMS itself, according to predefined ECA rules, and return the new parameters of the WfMS itself to SCP4WMS to be optimised with the new operating condition variables of the workflow engines.
  2.2. SOF4WMS should transmit the remaining parameters to SHM4WMS in order to deal with other WfMS mechanisms.
  2.3. SOF4WMS should verify the WfMS behaviour trustworthiness according to the algorithm for software/service behaviour trustworthiness validation and transform it into the subsequent mechanism SHM4WMS.
  2.4. When SOF4WMS receives the new parameters of the WfMS itself, modified by SHM4WMS, it should return these to SCP4WMS to be recovered with the new transaction consistence variables of the workflow engines.
3. SHM4WMS means self-healing-model system for the WfMS, and one of its functions is analysing the parameters transferred from the SOF4WMS component. According to the analysis, SHM4WMS carries out the following procedures.
  3.1. If the parameters of the transaction consistence variables of the workflow engines have changed and will cause an inconsistent transaction record in the WfMS, SHM4WMS will modify the parameters of the WfMS itself, according to predefined ECA rules, and return the new parameters to SOF4WMS to be recovered with the new transaction consistence variables of the workflow engines.
  3.2. SHM4WMS should compute the WfMS behaviour trustworthiness according to the NF algorithm (NF paradigms):
    3.2.1. If the WfMS NF is higher than or equal to the requirement NF of users when the ACM4WMS constructs the WfMS at the initial time, jump to step (3.3) directly.
    3.2.2. Otherwise, SHM4WMS will transfer it to the TWfMS method, which means that SHM4WMS will suggest the management and monitoring tool to transfer it to ACM4WMS through the local RE path (3.2.3). The management and monitoring tool transfers the NF that is lower than that of the initial WfMS to ACM4WMS through the local RE path.
  3.3. If ACM4WMS can reconstruct a WfMS with a new NF that is higher than or equal to the requirement NF of users from the source service in the cloud successfully, then jump to step (3.3) directly.
  3.4. Otherwise, the management and monitoring tool sends the NF that is lower than that of the initial WfMS to the ADCV-PN tools through the global RE path (that is, the ADCV-PN tools set will begin to remodel the WfMS under user validation of users).
  3.5. Register the new WfMS NF into the SHM4WMS database and return to step (1).

### 3.2. Measurement Algorithm for the Trustworthy Workflow Management System Based on Calculus

In this section, we introduce the general recursive measurement algorithm for the TWfMS based on the priority according to calculus. We provide the application server (AS) of the WfMS, consisting of the components and operations organised as a tree.

Firstly, the initial trustworthiness of AS is set to the value of 0.5, which means that we cannot judge whether or not it is more trustworthy than the initial value of 0.5 for proper trustworthiness when the ACM4WMS tool constructs the WfMS at the initial time, as illustrated in the following Equation (1):

(1) $$AS^{Initial\_Trustwothiness}=\langle C,O\rangle =0.5.$$

Secondly, we add the start and end components as the first and last activities of the original process with an absolute trustworthiness value of 1, which means they are coded with a simple but stable start or stop program with our absolute trust. Furthermore, we set the other components as the remaining immediate activities with an initial trustworthiness value of 0.5 for the same reason as that mentioned above. The initial trustworthiness of components is expressed by the following Equation (2):

(2) $$C=Components=\left\{\begin{array}{l} Original\_process_{Start}^{Initial\_Trustworthiness}=1,\\ Original\_process_{i,j,k\in [1,N]}^{Initial\_Trustworthiness}=0.5\\ Original\_process_{End}^{Initial\_Trustworthiness}=1 \end{array}\right\}.$$

Thirdly, we classify the operations among these components into three types of calculus. That is, for the operated component A, which is operated with the operating component B via dependency calculus C.

If, following the operation, the trustworthiness of A is replaced with the minimum trustworthiness of components A and B, we name C *strong dependency calculus*.

If, following the operation, the trustworthiness of A is replaced with the multiplication value of the trustworthiness of components A and B, we name C *indirect dependency calculus*.

If, following the operation, the trustworthiness of A is replaced with the average value of the trustworthiness of components A and B, we name C *weak dependency calculus*.

The operations can be illustrated as the following Equations: (3) with classes or (4) with a sequence order perspective from start, immediate, and end activities.

(3) $$O=Operations=\left\{\begin{array}{l} SDC^{Unsearched}=\{Strong\_Dependency\_Calculus^{Unsearched}\},\\ IDC^{Unsearched}=\{Indirect\_Dependency\_Calculus^{Unsearched}\},\\ WDC^{Unsearched}=\{Weak\_Dependency\_Calculus^{Unsearched}\}. \end{array}\right\}$$

(4) $$O=Operations=\{Calculus_{start,1}^{Unsearched};Calculus_{i,j}^{Unsearched};Calculus_{k,End}^{Unsearched}\}.$$

The trustworthiness of AS can be computed by the following Algorithm 1:
**Algorithm 1:** General_Recursive_Measure ($AS^{Initial\_Trustwothiness}$)Input: $AS^{Initial\_Trustwothiness}=0.5$; GRM=0.5; Output: $AS^{Trustwothiness}\in [0,1]$; 1: If AS.Components=∅; 2: Return; End if; 3: For every $Op_{i\in [start,1,2,\ldots ,n,end]}^{Initial\_Trustworthiness}$ do    //Traverse the tree in proper order of calculus priority from high to low:    $SDC^{Unsearched}>IDC^{Unsearched}>WDC^{Unsearched}$ 4: Compute behaviour trustworthiness ($Op_{i\in [start,1,2,\ldots ,n,end]}^{Trustworthiness}$) for $Op_{i\in [start,1,2,\ldots ,n,end]}^{Initial\_Trustworthiness}$, replace $Op_{i\in [start,1,2,\ldots ,n,end]}^{Initial\_Trustworthiness}$ with $Op_{i\in [start,1,2,\ldots ,n,end]}^{Trustworthiness}$ in $AS^{Initial\_Trustwothiness}$; 5: While exist $Calculus_{i,j}^{Unsearched}\in SDC^{Unsearched}$ do 6: *GRM* = General_Recursive_Measure ($Op_{j\in [start,1,2,\ldots ,n,end]}^{Initial\_Trustworthiness}$); 7: Replace $Calculus_{i,j}^{Unsearched}$ with $Calculus_{i,j}^{Searched}$ in $AS^{Initial\_Trustwothiness}$ 8: Set $Op_{i\in [start,1,2,\ldots ,n,end]}^{Trustworthiness}\leftarrow Op_{i\in [start,1,2,\ldots ,n,end]}^{Trustworthiness}\left(Calculus_{i,j}^{Searched}\right)GRM$; 9: End do; 10: While exist $Calculus_{i,j}^{Unsearched}\in IDC^{Unsearched}$ do 11: GRM = General_Recursive_Measure ($Op_{j\in [start,1,2,\ldots ,n,end]}^{Initial\_Trustworthiness}$); 12: Replace $Calculus_{i,j}^{Unsearched}$ with $Calculus_{i,j}^{Searched}$ in $AS^{Initial\_Trustwothiness}$ 13: Set $Op_{i\in [start,1,2,\ldots ,n,end]}^{Trustworthiness}\leftarrow Op_{i\in [start,1,2,\ldots ,n,end]}^{Trustworthiness}\left(Calculus_{i,j}^{Searched}\right)GRM$; 14: End do; 15: While exist $Calculus_{i,j}^{Unsearched}\in WDC^{Unsearched}$ do 16: GRM = General_Recursive_Measure ($Op_{j\in [start,1,2,\ldots ,n,end]}^{Initial\_Trustworthiness}$); 17: Replace $Calculus_{i,j}^{Unsearched}$ with $Calculus_{i,j}^{Searched}$ in $AS^{Initial\_Trustwothiness}$ 18: Set $Op_{i\in [start,1,2,\ldots ,n,end]}^{Trustworthiness}\leftarrow Op_{i\in [start,1,2,\ldots ,n,end]}^{Trustworthiness}\left(Calculus_{i,j}^{Searched}\right)GRM$; 19: End do; 20: End for; 21: Set $AS^{Init\_Trustwothiness}\leftarrow Op_{i\in [start,1,2,\ldots ,n,end]}^{Trustworthiness}$; 22: Replace $AS^{Init\_Trustwothiness}$ with $AS^{Trustwothiness}$; 23: Return $AS^{Trustwothiness}$.

Following completion of this algorithm, the trustworthiness of AS should be computed as follows:

(5) $$AS^{Trustwothiness}=\langle C,O\rangle \in [0,1].$$

(6) $$C=Components=\left\{\begin{array}{l} Original\_process_{Start}^{Trustworthiness}=1,\\ Original\_process_{i,j,k\in [1,N]}^{Trustworthiness}\in [0,1],\\ Original\_process_{End}^{Trustworthiness}=1. \end{array}\right\}$$

(7) $$O=Operations=\left\{\begin{array}{l} SDC^{Searched}=\{Strong\_Dependency\_Calculus^{Searched}\},\\ IDC^{Searched}=\{Indirect\_Dependency\_Calculus^{Searched}\},\\ WDC^{Searched}=\{Weak\_Dependency\_Calculus^{Searched}\}. \end{array}\right\}$$

(8) $$O=Operations=\{Calculus_{start,1}^{Searched};Calculus_{i,j}^{Searched};Calculus_{k,End}^{Searched}\}.$$

### 3.3. Basic Software Behaviour Trustworthiness Metric for Components

In this section, based on our prior works on data mining [20,21], and now in the uncertain environment, we introduce the basic component trustworthiness metrics expanded with POME in order to obtain the appropriate trustworthiness entropy of software behaviour according to the deterministic software behaviour of AS components.

**Definition** **1.** Trust is a three-tuple $\left(E_{1},E_{2},te_{E_{1}}^{E_{2}}\right)$, where $E_{1}$ is the trustor, $E_{2}$ is the trustee, $te_{E_{1}}^{E_{2}}$ is the value of trust entropy made by $E_{1}$ upon $E_{2}$, and $E1\cap E2=\emptyset ,E1\cup E2\neq \emptyset ;te_{E_{1}}^{E_{2}}\in [0,1]$.

**Definition** **2.** Software trustworthiness entropy (TE) is a combination entropy attribute consisting of sub-attributes according to the requirement, where TE∈[0,1] and a greater value of TE results in higher trust in the software.

**Definition** **3.** Software initialisation trustworthiness entropy ($T_{site}(s)$) is set at software start-up, where $T_{site}(s)\in [0,1]$ and greater values of $T_{site}(s)$ entropy mean higher trust in the initialised software is required.

**Definition** **4.** Software trusted threshold entropy ($T_{stte}(s)$) is set by the user prior to the software running, where $T_{stte}(s)\in [0,1]$ and greater values of $T_{stte}(s)$ entropy mean higher trust in the terminated software is required.

**Definition** **5.** Software runtime trustworthiness entropy ($T_{srte}(s)$) is measured at the software runtime by a software measurement tool or agent, according to its actual behaviour and user evaluation.

It is clear that the trustworthy software running condition should be $T_{site}(s)\geq T_{srte}(s)\geq T_{stte}(s)$; otherwise, the software should be terminated.

From the perspective of software engineering, all of the initial software attributes can be reflected by software test data entropy (STDE). It is well known that any STDE partition can be uniquely associated with an equivalence relation on the STDE. Therefore, we define the STDE as the static trustworthiness data to reflect the $T_{site}(s)$ through the equivalence partition of black-box testing prior to delivering the software. 

In contrast, all dynamic software attributes can only be reflected by software executed data entropy (SEDE). Thus, we define SEDE as dynamic trustworthiness data for reflecting the $T_{srte}(s)$ through an equivalence partition approach to black-box testing after delivering the software and comparison with the equivalence partition on STDE.

**Definition** **6.** Assume that X is an incomplete and finite collection consisting of STDE or SEDE, and recall that an equivalence relation R on X is a mapping R:X×X→{0,1}.

Therefore, we denote $R_{Ti}$ as test data when $R_{Ti}$ is a real case of R defined above and collected from a software test environment prior to being delivered for use.

In contrast, we denote $R_{Ei}$ as executed data when $R_{Ei}$ is a real case of R defined above and collected from a software runtime environment after being delivered for use.

**Definition** **7.** *Taking the equivalence relation R as rule-type information, according to the artificial intelligence theory, we can introduce the theory for software trustworthiness measurement and evaluation. Here, R is represented as follows:*

if R then H ; [0≤(CF(R),CF(R,H))≤1],

*where H means the trustworthiness of the owning trustee. The rule can be explained as follows: given that R occurred with a probability of CF(R), the trustee is the software itself, and the trustworthiness of the rule is (R,H) with probability CF(R,H). Thus, the trustworthiness of the software is H with probability CF(H).*

We can calculate *CF*(*H*) by means of criteria 1 to 3.

**Criterion** **1.** *According to the definitions above, CF(H) can be calculated as follows:*

(9) $$T_{site}(s)=CF(H)=CF(R,H)\times CF(R).$$

**Criterion** **2.** *Given an equivalence relation R on $X=\left\{x_{1},x_{2},\ldots \ldots ,x_{l}\right\}$, assume that we have two partitions of the test space X. According to the definition above, X comprises:*

(10) $$X=\overset{Card\left(\left\{\left(R_{i},H\right)\right\}\right)}{\underset{i=1}{\cup }}R_{i}.$$

(11) $$P_{STDE}=R_{T1},\ldots ,R_{Tp},R_{Ti}\cap R_{Tj}=\emptyset ,i\neq j,\overset{Tp}{\underset{i=1}{\cup }}R_{Ti}=STDE\subseteq X.$$

(12) $$P_{SEDE}=R_{E1},\ldots ,R_{Eq},R_{Ei}\cap R_{Ej}=\emptyset ,i\neq j,\overset{Eq}{\underset{i=1}{\cup }}R_{Ei}=SEDE\subseteq X.$$

**Criterion** **3.** *According to the definitions above, given an equivalence relation R on $X=\left\{x_{1},x_{2},\ldots \ldots ,x_{l}\right\}$, $n=Card\left(\left\{\left(R_{i},H\right)\right\}\right)$, CF(R) can be calculated as follows:*

(13) $$CF(R)=\sum_{i=1}^{n}\left(\sum_{j=1,x_{j}\in \left(R_{i},H\right)}^{l}pr\left(R_{i}|x_{j}\right)-\sum_{j=1,x_{j}\notin \left(R_{i},H\right)}^{l}pr\left(R_{i}|x_{j}\right)\right),$$

*where the uncertainty regarding CF(R) consisting of $pr\left(R_{j}|x_{i}\right)$, measured by the entropy function for $\forall x_{j}\in X$, $n=Card\left(\left\{\left(R_{i},H\right)\right\}\right)$, is given as follows:*

(14) $$\mathrm{Max}.\;\mathrm{size}:\;\underset{\forall x_{j}\in X}{\max}\left(-\sum_{i=1}^{n}\left[pr\left(R_{i}|x_{j}\right)\right]\ln\left[pr\left(R_{i}|x_{j}\right)\right]\right).$$

(15) $$\mathrm{Subject}\;\mathrm{to}:\;\sum_{i=1}^{n}\left[pr\left(R_{i}|x_{j}\right)\right]=1,\;pr\left(R_{i}|x_{j}\right)\geq 0.$$

(16) $$\sum_{i=1}^{n}\left[pr\left(R_{i}|x_{j}\right)\right]f_{k}\left(x_{k}\right)=E\left[f_{k}\right]=F_{k},\;k\in \left[1,m\right],$$

*where $pr\left(R_{i}|x_{j}\right)$ is the probability of each set of possible information or state $R_{i}$ related to evidence of whether or not the equivalence relation R belongs to user requirements, given every test item $x_{j}$ of X. Suppose that we obtain:*

(17) $$pr\left(R_{i}|x_{j}\right)=\frac{1}{Z\left(\lambda_{1},\lambda_{2},\ldots ,\lambda_{m}\right)}\exp\left[\lambda_{1}f_{1}\left(R_{i}\right)+\lambda_{2}f_{2}\left(R_{i}\right)+,\ldots ,+\lambda_{m}f_{m}\left(R_{i}\right)\right],$$

*where $Z\left(\lambda_{1},\lambda_{2},\ldots ,\lambda_{m}\right)=\sum_{i=1}^{n}\exp\left[\lambda_{1}f_{1}\left(R_{i}\right)+\lambda_{2}f_{2}\left(R_{i}\right)+,\ldots ,+\lambda_{m}f_{m}\left(R_{i}\right)\right]$, and the $\lambda_{k}$ parameters are Lagrange multipliers with values determined by $F_{k}=-\frac{\partial }{\partial \lambda_{k}}Z\left(\lambda_{1},\lambda_{2},\ldots ,\lambda_{m}\right)$.*

Above, we have introduced the definitions of STDE and SEDE associated with the $T_{site}(s)$ calculated on STDE. We consider formulating the congruence measurement from the perspective of the partitions on STDE and SEDE in order to calculate $T_{srte}(s)$.

It is critical to obtain a mapping congruence: $P_{STDE}\times P_{SEDE}\to \left[0,1\right]$ (where *P* stands for the equivalence relation on the software test data entropy (STDE) or software executed data entropy (SEDE)) indicating the degree of congruence or similarity between $P_{STDE}$ and $P_{SEDE}$.

Here, we calculate the congruence between $P_{STDE}$ and $P_{SEDE}$ using the underlying equivalence relations. We note that if, for x≠y, we indicate an unordered pair by 〈x,y〉, 〈x,y〉=〈y,x〉, and if X has $n=Card\left(\left\{\left(R_{i},H\right)\right\}\right)$ elements, we have $\left(\begin{array}{c} n\\ 2 \end{array}\right)=\frac{\left(n)(n-1\right)}{2}=n^{c}2$ unordered pairs.

We now suggest a general congruence measure between partitions of $P_{STDE}$ and $P_{SEDE}$, which we express in terms of their underlying equivalence relations.

(18) $$\bar{Cong}(P_{STDE},P_{SEDE})=1-\frac{Diff\_Val(P_{STDE},P_{SEDE})}{n^{c}2},$$

where $D=Diff\_Val(P_{STDE},P_{SEDE})$ is the number of pairs that have different values in $P_{STDE}$ and $P_{SEDE}$. Then, we can calculate the software runtime trustworthiness $T_{srte}(s)$ from the software initialization trustworthiness $T_{site}(s)$:

(19) $$T_{srte}(s)=\bar{Cong}(P_{STDE},P_{SEDE})\times T_{site}(s).$$

In the section above, we have introduced a general measure of similarity or congruence between two partitions on STDE and SEDE using the underlying equivalence relations. Equation (19) implies that we should traverse all of the equivalence relations from the STDE and SEDE circularly. Thus, the largest complexity of Equation (19) is $O\left({\left(Card\left(R_{i})|\left(R_{i},H\right)\in X\right)\right)}^{2}\right)$.

We now consider the perspective of the partitions themselves. Taking into account Equations (11) and (12), without loss of generality, we can assume that q=p, and if q>p, we can augment the partition $P_{SEDE}$ by adding q−p subsets $R_{Ep+1}=R_{Ep+2}=\ldots =R_{Eq}=\emptyset$. Thus, in the following we assume that the two partitions have the same number of classes, q. We now introduce an operation known as a pairing of $P_{STDE}$ and $P_{SEDE}$, denoted by $g\left(P_{STDE},P_{SEDE}\right)$, which associates with each subset $R_{Ti}$ of $P_{STDE}$ a unique $R_{Ei}$ from $P_{SEDE}$, grouped according to $\left(R_{i},H\right)\in X$. We then have the fact that a pairing $g\left(P_{STDE},P_{SEDE}\right)$ is a collection of q pairs, $g\left(P_{Ti},P_{Ei}\right)$. We now associate with each pairing a score, $g\left(P_{STDE},P_{SEDE}\right)$, defined as follows. Denoting $D_{g.j}=P_{Tj}\cap P_{Ej}$, for j=1 to q, we obtain:

(20) $$Score\left(g\left(P_{STDE},P_{SEDE}\right)\right)=\sum_{j=1}^{q}Card\left(D_{g.j}\right).$$

We now use this to obtain the congruence:

(21) $$\bar{\bar{Cong}}(P_{STDE},P_{SEDE})=\frac{Score\left(g\left(P_{STDE},P_{SEDE}\right)\right)}{Card(X)}.$$

Then, we can calculate $T_{srte}(s)$ from $T_{site}(s)$,

(22) $$T_{srte}(s)=\bar{\bar{Cong}}(P_{STDE},P_{SEDE})\times T_{site}(s).$$

Through analysis, the complexity of Equation (22) is determined as O(Card(STDE)×Card(SEDE)), which is less than or equal to the complexity of $\bar{Cong}(P_{STDE},P_{SEDE})$ in Equation (18), because Card(STDE)≤Card(X) and Card(SEDE)≤Card(X), according to Equations (6) and (7).

Therefore, can we conclude that the performance of Equation (22) is far superior to that of Equation (19) simply by their differing complexities? Indeed, with the trends of infrastructure-as-a-service (IaaS), platform-as-a-service (PaaS), and software-as-a-service (SaaS) in the cloud, increasing software components encapsulated as services are emanating from third parties so that there no longer exists a steady and closed STDE. For this reason, the precondition of Equation (11) that clusters STDE into the test space X would visibly increase its complexity.

## 4. Conclusions

In order to address the measurement problem of WfMS trustworthiness, based on prior research [17,18,19,20,21], this paper has proposed a novel algorithm for WfMS trustworthiness based on the TWfMS framework mechanisms in an uncertain environment with incomplete software behaviour test cases, which means that the deterministic entropy of services or its underlying software behaviour is partial. Similar to BPM, we can consider the entire WfMS lifecycle as a group of long-term processes, which we categorise into three aspects: the ‘as-is process’ in the build-time stage, the ‘to-be process’ in the runtime stage, and the ‘agile-consistent process’ in maintenance time. This study focuses on the measurement algorithm of the first SCP4WMS mechanism of the ‘agile-consistent process’, which supports the computing infrastructure of the SOF4WMS and SHM4WMS mechanisms. In order to guarantee the current agile and consistent attributes in the WfMS mechanisms, we serialise the three mechanisms SCP4WMS, SOF4WMS, and SHM4WMS in a sequence list to solve the agile and consistent WfMS problems into three self-autonomous propriety grades: functionalities, non-functionalities, and transactions. Direct feedback is used to adjust the workflow engine online by the TDC, accompanied by three trustworthy propriety grades–measurement, verification, and evaluation—indirectly proposed for the ACM4WMS or ADCV-PN tools by the management and monitoring tool encountered in the LRE or GRE loops. Moreover, this means that, for exception events that cannot be solved by WfMS mechanisms, we transform these with event parameters into the method of the WfMS in order to pursue further solving with LRE or GRE. 

In summary, our study is closely related to former works, but it differs from these in terms of two aspects on which we place emphasis simultaneously: self-autonomic computing and trust computing, including the evaluation method [22], which indicates that Equation (13), if applied only to the CF(R) of Equation (9) and not the CF(R,H), has not resolved the problem of incomplete information on the set of (R,H). Similarly, Equations (18) and (19) are all based on the relatively static software test environment while WfMSs are in runtime. Then, we compare the difference between the $P_{STDE}$ and $P_{SEDE}$ by using Equation (18) and compute the $T_{srte}(s)$ by using Equation (19). Indeed, in order to obtain more precise $T_{site}(s)$ and $T_{srte}(s)$, we might update Equations (18) and (19) with the same style as Equation (9) in future works. Furthermore, we plan to implemented the WfMS based on the fundamental features of Internetware [23], and all of these works involve software architecture or service paradigms representing their trustworthiness concentrically, from direct or indirect viewpoints, which also indicate that the $P_{SEDE}$ would be more frequently influenced by the dynamic software test environment while WfMSs are more in runtime than the $P_{STDE}$, which is generated from the relatively static software test environment.

In future work, we plan to conduct simulations or practical industry experiments to verify and evaluate our measurement algorithm for the WfMS mechanism. It is our hope that future works will be completed in the context of the reduction process of WfMS behaviour entropy, given the unavoidable nature of WfMS behaviour entropy increasing.
